# Correlates of nonadherence to key population-led HIV pre-exposure prophylaxis services among Thai men who have sex with men and transgender women

**DOI:** 10.1186/s12889-019-6645-0

**Published:** 2019-03-21

**Authors:** Pich Seekaew, Ezie Nguyen, Thanthip Sungsing, Jureeporn Jantarapakde, Supabhorn Pengnonyang, Deondara Trachunthong, Pravit Mingkwanrungruang, Waraporn Sirisakyot, Pattareeya Phiayura, Phubet Panpet, Phathranis Meekrua, Nanthika Praweprai, Fonthip Suwan, Supakarn Sangtong, Pornpichit Brutrat, Tashada Wongsri, Panus Rattakittvijun Na Nakorn, Stephen Mills, Matthew Avery, Ravipa Vannakit, Praphan Phanuphak, Nittaya Phanuphak

**Affiliations:** 1 PREVENTION, the Thai Red Cross AIDS Research Centre, 104 Rajdamri Rd., Pathumwan, Bangkok, 10330 Thailand; 2 Keck School of Medicine, University of Southern California, Los Angeles, California USA; 3 Rainbow Sky Association of Thailand, Bangkok, Thailand; 4 Service Workers IN Group (SWING) Foundation, Bangkok, Thailand; 5 Sisters Foundation, Pattaya, Chonburi Thailand; 6 Rainbow Sky Association of Thailand, Songkhla, Thailand; 7 MPlus Foundation, Chiang Mai, Thailand; 8 SWING Foundation, Pattaya, Chonburi Thailand; 9 Caremat Foundation, Chiang Mai, Thailand; 10 FHI 360 and LINKAGES Project, Bangkok, Thailand; 11 Office of Public Health, U.S. Agency for International Development Regional Development Mission for Asia, Bangkok, Thailand

**Keywords:** HIV prevention, MSM, PrEP adherence, Key population-led, Pre-exposure prophylaxis, Transgender women

## Abstract

**Background:**

Based on government estimates from the Asian Epidemic Model, new infections among men who have sex with men (MSM) and transgender women (TGW) in Thailand are forecast to proportionally increase over time. Daily oral Pre-exposure prophylaxis (PrEP) protects against HIV acquisition when used as prescribed. The “Princess PrEP” program is the first key population-led (PrEP) initiative under Thai royal patronage with an aim to scale up countrywide implementation of PrEP.

**Methods:**

Retention in and adherence to key population-led HIV PrEP services among HIV-uninfected Thai MSM and TGW was examined in four provinces: Bangkok, Chonburi, Chiang Mai, and Songkhla. HIV, HBsAg, creatinine tests, and self-administered questionnaires were performed during baseline measures. Participants were followed up after month 1, at month 3, then every 3 months. Correlates of nonadherence and loss to follow up at 1 month were assessed using linear regression models.

**Results:**

37.4% of the participants reported low adherence to services (≤ 3 pills/week or missed clinic schedule at month 1). Factors associated with low adherence included younger age (25 years and under) (adjusted odds ratio (aOR): 1.49, 95% confidence interval (95% CI: 1.01–2.21, *p* = 0.044), being a TGW (aOR: 2.2, 95% CI: 1.27–3.83, *p* = 0.005), and whether the participant had not previously accessed services at the clinic (aOR = 1.68, 95% CI: 1.03–2.76, *p* = 0.04). Additionally, participants in Chonburi (the only TGW site) showed significantly lower adherence than those in the other three provinces (aOR: 2.91, 95% CI: 1.55–5.45, *p* = 0.001).

**Conclusion:**

Urgent, innovative interventions for early PrEP adherence support among vulnerable sub-populations such as younger users, TGW, and new clients are needed to maximize prevention strategy in Thailand.

**Electronic supplementary material:**

The online version of this article (10.1186/s12889-019-6645-0) contains supplementary material, which is available to authorized users.

## Background

Throughout the past decade, Thailand has successfully continued to reduce their HIV epidemic [1]. Concerted efforts countrywide have decreased the total number of annual new infections from 10,215 in 2010 to 7816 in 2014 [2]. Despite Thailand’s overall progress, not all key populations such as men who have sex with men (MSM) and transgender women (TGW) reflect the same improvement. Approximately 44.4% of new infections from 2012 to 2016 were attributed to MSM, TGW, and male sex workers [3]. Based on government estimates from the Asian Epidemic Model, new infections among these populations are forecast to proportionally increase over time making them a priority for HIV prevention work [4].

Multiple studies have shown that daily oral tenofovir disoproxil fumarate and emtricitabine (TDF/FTC) in the pill form prevents HIV acquisition in individuals at higher risk of HIV acquisition when used as pre-exposure prophylaxis (PrEP) [5, 6]. As a response to the disproportionate HIV burden for MSM and TGW, Thailand has introduced various prevention initiatives including reduced-cost and free PrEP service programs [7–9]. The “Princess PrEP” program is a demonstration project initiated in 2016 by the Thai Red Cross AIDS Research Centre (TRCARC) and funded by the Princess Soamsawali Fund for HIV Prevention and the LINKAGES program, funded through the US President’s Emergency Fund for AIDS Relief (PEPFAR) through USAID, and managed by FHI 360 [7]. The program is the first key population-led PrEP initiative under royal patronage with an aim to assist in scaling up a countrywide implementation of PrEP. This initiative utilizes a Key Population-Led Health Service (KPLHS) delivery model intending to provide PrEP for 3000 MSM and TGW over 3 years [7]. KPLHS are a defined set of HIV-related health services that focus on specific key populations and are delivered by community-based organizations (CBOs) run by those same key populations in partnership with other health sector entities. In this context, community leadership means that the services necessary for addressing the HIV epidemic and related health issues are identified by the community itself and are, therefore, needs-based, demand-driven, and client-centered.

Under Princess PrEP, free rapid HIV testing and PrEP are provided by trained key population community health workers (KP-CHW) at seven Thai CBOs in Bangkok, Chonburi, Chiang Mai, and Songkhla provinces. The program has had success in enrollment; however, there are issues regarding retention in care and adherence to services.

PrEP has been clinically proven to reduce HIV transmission [5, 6]; however, studies indicate that the efficacy of PrEP is strongly associated with user adherence [10, 11]. Adherence can be defined as the degree to which PrEP users follow their prescribed once-daily dosage schedule [12]. In a study, PrEP users who took their medication at least four times a week had drug concentrations that resulted in 90% drug efficacy or higher [13]. Some empirical research provides greater insight into correlates of those that may be more susceptible to nonadherence, but these findings are limited to specific demographics and a clinical setting [12, 14, 15]. The effectiveness and feasibility of PrEP implementation in real-world settings from limited-resource, middle income countries are widely unknown. Additionally, no reliable data are available about PrEP adherence patterns among Thai MSM and TGW, resulting in questions on how to increase adherence and retention to HIV prevention care among these populations.

This study examined the correlates of the participants’ adherence patterns, demographics, and sexual risk behaviors at month 1. Inferences were drawn from month 1 data, as to identify groups susceptible to nonadherence and those who may benefit from additional adherence and retention support early on in their care. A study examining adherence to PrEP among adolescent MSM detected a decreasing trend in adherence over time [15]. This analysis aimed to assess factors associated with nonadherence and loss to follow up in order to optimize support in the “Princess PrEP” and other initiatives similar in nature.

## Methods

### Participants and procedures

Participants were enrolled into the Princess PrEP program over the course of 13 months (January 2016–February 2017) at the seven participating community health centers run by CBOs. These CBOs are Rainbow Sky Association of Thailand (RSAT) serving MSM and TGW in Bangkok and Songkhla, Service Workers in Group (SWING) serving male sex workers and MSM in Bangkok and Chonburi, Sisters serving transgender women sex workers and TGW in Chonburi, and Caremat and MPlus serving MSM and TGW in Chiang Mai. To be eligible for the program, participants must be self-identified as MSM or TGW, reported recent (within the past 3 months) and current HIV risk factors as assessed by KP-CHW, and agreed to use PrEP as part of the combination HIV prevention package. There was no lower age limit to access PrEP in this program. During baseline measures, all questionnaires were self-administered and HIV, HBsAg, and creatinine tests were performed. Participants were followed up after month 1, at month 3, then every 3 months. Additional HIV tests were performed at every visit and creatinine was measured every 6 months. The follow up for month 1 was arranged during the baseline visit. Clients were reminded of their appointment 2 weeks in advance via a mobile online application (e.g. LINE and Facebook). Participants who missed their appointment were called for follow-up every week for 1 month then once every month. Participants unable to be reached were labeled as “No Show”. Analyses were drawn from month 1 measures and compared to baseline measures. The program was approved by the institutional review board of Chulalongkorn University. All participants gave verbal informed consent.

### Measures

The survey queried participant demographics including age as a continuous variable, gender identity (male or TGW), nationality (Thai or non-Thai), marital status, education, occupation, and income. Participants indicated their current sexual risk factors, including accessing HIV testing and services at the clinic previously, age at first intercourse, and perceived HIV risk, in addition to their sexual risk factors within the past 3 months—number and gender of sexual partners, condom usage, substance usage, symptoms and/or diagnoses of sexually transmitted diseases (gonorrhea, chlamydia, herpes, or syphilis), and group sex behavior (frequency, condom usage, and drug usage) [See Additional file 1].

### Data analysis

All statistical analyses were conducted using Stata version 15 software (StataCorp LLC, College Station, Texas). The demographic characteristics of the participants together with their baseline behavior risk information were examined overall and by subgroups of PrEP adherence. Based on previous literature constituting proper PrEP usage and efficacy, PrEP adherence was categorized into 2 levels: Low Adherence (no follow-up or ≤ 3 pills/week) and Good Adherence (≥ 4pills/week). For categorical parameters, frequency and proportion were reported. Mean with standard deviation (SD) and median with interquartile range (IQR) were reported for continuous parameters. Comparisons of continuous variables between groups of PrEP adherence were made by using independent samples t-test or Wilcoxon Rank Sum Test. Bivariate chi-square tests were performed comparing categorical factors for demographics and risk behaviors between adherence groups. Fisher’s Exact test was performed in cases where expected cell count less than five was greater than 20% of total number of cells.

Binary logistic regression was performed to identify factors associated with PrEP adherence. Binary outcome was categorized by PrEP subgroups. Statistically significant variables at the univariate *p* < 0.15 level and those that were clinically significant were subsequently adjusted in the multivariable logistic regression model by enter method.

## Results

There were 829 participants enrolled in the Princess PrEP program during the study period. Of these, 72 (8.7%) have not reached the first month schedule before analysis and data on PrEP adherence were missing for 104 (12.5%) participants who were followed up at month 1. The final analytical sample size included 564 MSM and 89 TGW.

### Participant sociodemographic and sexual risk behavior

Of 653 participants, 244 (24 reported taking ≤3 PrEP pills per week and 220 did not show up at month 1) were categorized as having low adherence (37.4%). Furthermore, 480 (73.5%) of the participants were recruited from Bangkok, 112 (17.2%) from Chonburi, 36 (5.5%) from Chiang Mai, and 25 (3.8%) from Songkhla. From the provinces listed, Chonburi contains the only stand-alone health center exclusively for TGW. And since this is part of the service, there is no predetermined quota on how many participants each site could enroll, or the proportion between MSM and TGW, resulting in variations in the number of participants. Table 1 reports sociodemographic information and their associations with PrEP adherence. The mean (SD) age of the sample was 28.9 (7.3) years, with participants designated as having low adherence significantly younger than those who had good adherence (27.8 vs. 29.5 years, *p* = 0.0027). Low adherence was reported more frequently by TGW than MSM (65.2% vs. 32.97%, *p* < 0.001), participants in Chonburi than other provinces (66.1% vs. 31.4%, *p* < 0.001), non-Thai participants than Thais (59.4% vs. 36.3%, *p* = 0.009), participants with less than a bachelor’s degree than those with higher degrees (52.5% vs. 28.6%, *p* < 0.001), service workers than others (62.1% vs. 33.6%, *p* < 0.001), and participants with monthly income lower than 20,000 Thai Baht than those with higher income (42.6% vs. 30.0%, *p* = 0.014).

**Table 1:** Bivariate associations of participant sociodemographics and adherence subgroups among MSM and TGW in the Princess PrEP program (*N* = 653)

Variables	Total *N* (%)	Low Adherence: ≤ 3 Pills per week or no show at Month 1 N (%)	Good Adherence: 4–7 Pills per Week N (%)	*p*-value
Total	653 (100)	244 (37.4)	409 (62.6)	
Age (years)				
Mean (SD)	28.9 (7.3)	27.8 (7.3)	29.5 (7.1)	0.003^a^
Age ≤ 25	245 (37.5)	110 (44.9)	135 (55.1)	0.002^c^
Age > 25	408 (62.5)	134 (32.8)	274 (67.2)	
Gender				< 0.001^c^
Male	564 (86.4)	186 (33.0%)	378 (67.0)	
TGW	89 (13.6)	58 (65.2)	31 (34.8)	
Sites				< 0.001^c^
Bangkok	480 (73.5)	151 (31.5)	329 (68.5)	
Chonburi	112 (17.2)	74 (66.1)	38 (33.9)	
Chiang Mai	36 (5.5)	6 (16.7)	30 (83.3)	
Songkhla	25 (3.8)	13 (52.0)	12 (48.0)	
Nationality				0.009^c^
Thai	609 (93.3)	221 (36.3)	388 (63.7)	
Non-Thai	32 (4.9)	19 (59.4)	13 (40.1)	
Marital status				0.66^d^
Single	564 (86.4)	214 (38.0)	350 (62.1)	
Living together/Married	64 (9.8)	24 (37.5)	40 (62.5)	
Divorced/Widow	6 (0.9)	1 (16.7)	5 (83.3)	
Education				< 0.001^c^
Less than Bachelor degree	242 (37.1)	127 (52.5)	115 (47.5)	
Bachelor degree and above	374 (57.3)	107 (28.6)	267 (71.4)	
Occupation				< 0.001^c^
Unemployed/Student	119 (18.2)	42 (35.3)	77 (64.7)	
Employed	435 (66.6)	144 (33.1)	291 (66.9)	
Service Worker (beautician, restaurant, etc.)	87 (13.3)	54 (62.1)	33 (37.9)	
Monthly Income (THB)				0.014^c^
Less than or equal 10,000 THB	100 (15.3)	45 (45.0)	55 (55.0)	
10,001–20,000 THB	243 (37.2)	101 (41.6)	142 (58.4)	
20,001–50,000 THB	171 (26.2)	48 (28.1)	123 (71.9)	
Greater than 50,000 THB	39 (6)	15 (38.5)	24 (61.5)	
Have ever had HIV testing/services at clinic before enrollment in this study				0.17^c^
New Client	539 (82.5)	208 (38.6)	331 (61.4)	
Yes	102 (15.6)	32 (31.4)	70 (68.6)	

^a^Student’s t-tests/Unpaired t-test

^b^Mann-Whitney/Wilcoxon rank-sum test

^c^Chi square test

^d^Fisher’s Exact test

*MSM* men who have sex with men, *TGW* transgender women, *THB* Thai Baht

In the past 3 months, inconsistent condom use during anal sex was reported by 276 participants (42.3%), median (IQR) number of male sex partners was 3 (2–8), 37 (5.7%) used amphetamine-type stimulants, 131 (20.1%) had group sex. During group sex, 43 (32.8%) inconsistently used condom and 64 (48.8%) used drugs. Table 2 lists bivariate correlates of sexual risk behavior and PrEP adherence. Participants with low adherence had higher number of male sex partners in the past 3 months (4 vs. 3, *p* = 0.008).

**Table 2:** Bivariate associations of participant sexual risk behavior and adherence subgroups among MSM and TG (*N* = 653)

Variables	Total N (%)	Low Adherence: ≤ 3 Pills per week or no show at month 1 N (%)	Good Adherence: 4–7 Pills per week N (%)	*p*-value
Age at first sexual intercourse (years)				
Median (IQR)	18 (16–21)	18 (15–20)	18 (16–21)	0.12^a^
< 18	220 (33.7)	86 (39.1)	134 (60.9)	0.19^b^
≥ 18	376 (57.6)	127 (33.8)	249 (66.2)	
Sexual partners in lifetime				
Male only	432 (66.2)	153 (35.4)	279 (64.6)	
TGW only	15 (2.3)	12 (80.0)	3 (20.0)	
Male & Female	116 (17.8)	37 (31.9)	79 (68.1)	
Male & TGW	6 (0.9)	3 (50.0)	3 (50.0)	
Female & TGW	5 (0.8)	1 (20.0)	4 (80.0)	
Male/Female/TGW	19 (2.9)	6 (31.6)	13 (68.4)	
HIV perceived risk in the past 3 months				0.38^b^
No risk	59 (9)	27 (45.8)	32 (54.2)	
Mild	217 (33.2)	75 (34.6)	142 (65.4)	
Moderate	222 (34)	81 (36.5)	141 (63.5)	
High	109 (16.7)	36 (33.0)	73 (67.0)	
Had sexual intercourse with male partner in past 3 months	581 (89)	209 (36.0)	372 (64.0)	0.61^b^
Median (IRQ) of male partners	3 (2–8)	4 (2–10)	3 (2–5)	0.008^a^
Had sexual intercourse with female partner in past 3 months	43 (6.6)	20 (46.5)	23 (53.5)	
Median (IRQ) of female partners	1 (1–3)	2 (1–3)	1 (1–3)	
Had sexual intercourse with TGW partner in the past 3 months	16 (2.5)	7 (43.8)	9 (56.2)	
Median (IRQ) of TG partners	1 (1–3)	1.5 (1–4)	1 (1–2.5)	
Number of sexual partners in the past 3 months (all MSM, female and TGW)				0.09^b^
No sexual partner	17 (2.6)	8 (47.1)	9 (52.9)	
Single partner	122 (18.7)	40 (32.8)	82 (67.2)	
Multiple partners	418 (64.0)	146 (34.9)	272 (65.1)	
Had sexual intercourse in the past 3 months but didn’t specify number of sex partners	51 (7.8)	26 (51.0)	25 (49.0)	
Number of sex partners in past 3 months (all MSM, female and TGW) (median, IQR)	3.5 (2–8)	4 (2–10)	3 (2–6)	0.006^a^
Condom use (anal receptive or insertive) in the past 3 months				0.42^b^
Protected sex (No sexual activity + Always)	285 (43.6)	96 (33.7)	189 (66.3)	
Unprotected sex (Never + Sometimes)	276 (42.3)	102 (37.0)	174 (63.0)	
Substance/stimulant/drug use in the past 3 months				0.98^b^
No	374 (57.3)	134 (35.8)	240 (64.2)	
Yes (Overall participants that reported substance/stimulant/drug use at least once)	221 (33.8)	79 (35.7)	142 (64.3)	
If “Yes”, substance/stimulant/drug use in the past 3 months				
Alcohol	128 (19.6)	48 (37.5)	80 (62.5)	
Methamphetamine/amphetamine	33 (5.1)	12 (36.4)	21 (63.6)	
Ecstasy	7 (1.1)	3 (42.9)	4 (57.1)	
Drug Application (Ketamine).	6 (0.9)	4 (66.7)	2 (33.3)	
Poppers	82 (12.6)	20 (24.4)	62 (75.6)	
Cocaine	5 (0.8)	4 (80.0)	1 (20.0)	
Marijuana	7 (1.1)	3 (42.9)	4 (57.1)	
Barbiturates or other sedatives	1 (0.2)	1 (100.0)	0 (0)	
Viagra or other drugs in same group	59 (9.0)	20 (33.9)	39 (66.1)	
Amphetamine-type stimulant used in past 3 months				0.83^b^
No	571 (87.4)	206 (36.1)	365 (63.9)	
Yes	37 (5.7)	14 (37.8)	23 (62.2)	
Symptoms of STI or previously diagnosed with STI (gonorrhea, chlamydia, herpes, syphilis) in the past 3 months				0.67^b^
None	472 (72.3)	170 (36.0)	302 (64.0)	
Yes, have any STI	68 (10.4)	22 (32.4)	46 (67.6)	
Unsure	47 (7.2)	19 (40.4)	28 (59.6)	
Had group sex in the past 3 months				0.44^b^
No	435 (66.7)	150 (34.5)	285 (65.5)	
Yes	131 (20.1)	50 (38.2)	81 (61.8)	
Yes, Median (IQR) # of times of group sex	3 (1–3)	2.5 (2–3)	3 (1–3)	0.88^a^
Yes, Median (IQR) of # of partners during group sex each time	3 (3–3)	3 (3–3)	3 (3–3)	0.89^a^
If yes, use of condom during group sex				0.09^c^
No	5 (3.8)	4 (80.0)	1 (20.0)	
Sometimes	38 (29.0)	16 (42.1)	22 (57.9)	
Always	80 (61.1)	27 (33.8)	53 (66.3)	
If yes, use of stimulants/drugs before/during group sex				0.34^b^
No	61 (46.6)	25 (41.0)	36 (59.0)	
Sometimes	46 (35.1)	18 (39.1)	28 (60.9)	
Always	18 (13.7)	4 (22.2)	14 (77.8)	

^a^Mann-Whitney/Wilcoxon rank-sum test

^b^Chi square test

^c^Fisher’s Exact test

*IQR* interquartile range, *MSM* men who have sex with men, *TGW* transgender women, *STI* sexually transmitted infection

### Multivariable correlates of PrEP adherence

Table 3 presents multivariable associations of sociodemographic information and baseline risk factors with PrEP adherence. After adjusting for multicollinearity (i.e. removing factors education and income) in the model, participants younger than 25 were significantly more likely to have low adherence to PrEP (adjusted odds ratio (aOR): 1.49, 95% confidence interval (95% CI): 1.01–2.21, *p* = 0.044). TGW had greater odds of having low PrEP adherence than MSM (aOR: 2.2, 95% CI: 1.27–3.83, *p* = .005). Participants in Chonburi were significantly more likely to have low PrEP adherence (aOR: 2.91, 95% CI: 1.55–5.45, *p* = 0.001) than those in other provinces. Finally, new clients had higher odds of having low PrEP adherence compared to those who reported having HIV testing or services at the community health center before enrollment in the program (aOR = 1.68, 95% CI: 1.03–2.76, *p* = 0.04).

**Table 3:** Binary logistic regression of factors independently associated with low adherence (< 3 pills per week or no show at month 1) among Thai MSM and TGW (*N* = 653)

Variables	Univariate			Multivariable		
	aOR	95% CI	*p*	aOR	95% CI	*p*
Demographic data						
Age (years)			0.002			0.044
Age ≤ 25	1.67	1.20–2.31		1.49	1.01–2.21	
Age > 25	Ref	–		Ref	–	
Gender			< 0.001			0.005
TGW	3.8	2.38–6.08		2.2	1.27–3.83	
Male	Ref	–		Ref	–	
Sites			< 0.001			
Chiang Mai/Songkhla	0.99	0.55–1.75		0.97	0 .53–1.79	0.924
Chonburi	4.24	2.74–6.56		2.91	1.55–5.45	0.001
Bangkok	Ref	–		Ref	–	
Nationality			0.01			0.28
Thai	Ref	–		Ref	–	
Foreigner	2.57	1.24–5.30		1.57	0.70–3.52	
Marital status			0.52			
Single	Ref	–				
Living together/Married	0.98	0.58–1.67				
Divorced/Widow	0.33	0.04–2.82				
Education*			< 0.001			
Less than Bachelor degree	2.76	1.97–3.86				
Bachelor degree and above	Ref	–				
Occupation*			< 0.001			
Unemployed/Student	1.1	0.72–1.69		0.93	0.57–1.52	0.76
Employed	Ref	–		Ref	–	
Service Worker (beautician, restaurant, etc.)	3.31	2.05–5.33		1.09	0.57–2.10	0.92
Income (THB)*			0.0029			
Less than or equal 20,000 THB	1.73	1.20–2.49				
Greater than 20,000 THB	Ref	–				
Have ever had HIV testing/services at clinic before enrollment in this study			0.16			0.04
New Client	1.37	0.87–2.16		1.68	1.03–2.76	
Yes	Ref	–		Ref	–	
Risk behavior at baseline						
Age at first sexual intercourse (years)			0.19			
< 18	1.26	0.89–1.78				
≥ 18	Ref	–				
HIV perceive risk in the past 3 months			0.39			
No risk	1.71	0.89–3.28				
Mild	1.07	0.66–1.74				
Moderate	1.16	0.72–1.89				
High	Ref	–				
Number of sexual partners in the past 3 months (all MSM, female and TG)			0.93			
No sexual partner/Single partner	Ref	–				
Multiple partners	1.02	0.68–1.52				
Number of sex partners in past 3 months (all MSM, female and TG)			0.27			
< 3	Ref	–				
≥ 3	1.23	0.85–1.80				
Condom use (anal receptive or insertive) in the past 3 months			0.42			
Safe sex (No sexual activity + Always)	Ref	–				
Unprotected sex (Never + Sometimes)	1.15	0.82–1.63				
Substance/stimulant/drug use in the past 3 months			0.98			
No	Ref	–				
Yes (Overall participants that reported substance/stimulant/drug use at least once)	1	0.70–1.41				
Amphetamine-type stimulant use in the past 3 months			0.83			
None	Ref	–				
Yes	0.93	0.47–1.84				
Symptoms of STI or previously diagnosed with STI (gonorrhea, chlamydia, herpes, syphilis) in the past 3 months			0.67			
None	Ref	–				
Yes, have any STI	0.85	0.49–1.46				
Unsure	1.21	0.65–2.22				
Had group sex in the past 3 months			0.61			
Didn’t have group sex	Ref	–				
Yes, times of group sex < 3	1.27	0.56–2.89				
Yes, times of group sex ≥3	1.15	0.74–1.79				
If yes, number of partners during group sex			0.73			
Didn’t have group sex	Ref	–				
Yes, # of sex partners during group sex each time < 3	1.27	0.56–2.89				
Yes, # of sex partners during group sex each time ≥ 3	1.15	0.74–1.79				
If yes, condom usage during group sex			0.29			
Didn’t have group sex	Ref	–				
None or Sometimes	1.65	0.88–3.11				
Always	0.97	0.58–1.60				
If yes, substance usage during group sex			0.61			
Didn’t have group sex	Ref	–				
Never	1.32	0.76–2.28				
Sometimes or Always	1	0.57–1.73				

*Education, occupation, and income were found to have collinearity. Occupation was chosen to include into the final logistic model

## Discussion

As countrywide PrEP implementation is underway in Thailand, adherence and retention support for PrEP users will be essential in advancing the country’s approach to providing effective HIV prevention to key populations. This study examined the characteristics of MSM and TGW PrEP users in the Princess PrEP program, which is the first key population-led PrEP initiative in Thailand, and provided insight on groups that may be susceptible to low PrEP adherence or care retention. More than one third of participants (37.4%) were loss to follow up or categorized as having low adherence or which further supports the urgent need to identify innovative ways to provide PrEP adherence and care retention support to MSM and TGW in the country. Among those categorized as having low adherence to services, approximately 90% were given this categorization because they did not attend the first follow up visit. Meanwhile, over 94% of those who did report for the follow up visit at month 1 were categorized as having good adherence. Therefore, efforts should largely emphasize strengthening program retention, rather than medication adherence, among users. The findings of this study remain consistent with literature on factors associated with poor HIV medication adherence in MSM and TGW, such as younger age, lower socioeconomic status, lower education, and increased sexual risk behaviors [12, 14].

The results chiefly underscore the need to prioritize PrEP implementation programs. Currently, there are no PrEP services integrated into Thailand’s national healthcare schemes, albeit demonstration projects are ongoing [16]. To address difficulties in retention in populations at higher risk of HIV acquisition, recognition and endorsement of PrEP as a key preventative modality in the national guidelines is warranted, as to increase resources and support for behavioral research in this area. Specifically, the findings highlight the potential for greater governmental involvement at the community and policy levels.

In the multivariable analysis, age was statistically significantly associated with lost retention or low adherence to PrEP programs suggesting younger users may benefit from additional support. Especially provided that young MSM reported higher HIV incidence rates from 2006 to 2014 compared to other key populations, targeted support could considerably impact this sub-population [2]. Young Thai MSM and TGW constitute a large proportion of internet and technology users in Thailand [17, 18]. Furthermore, data shows that this population strongly prefers online-to-offline healthcare models [19]. Adapting existing, efficacious offline healthcare support for online platforms is an initial step towards potentially improving retention and adherence in PrEP programs. This strategy has proven feasible and effective in both reducing barriers towards HIV medication adherence in youth living with HIV and treatment linkage in MSM [20, 21]; however, these models have not been optimized for models using PrEP. For example, findings from a pilot study suggested that LifeSteps, an offline cognitive behavioral intervention, integrated into PrEP programs effectively optimized adherence in MSM [22]. Transitioning such interventions onto an online platform could improve PrEP retention and adherence among young Thai MSM and TGW through decreasing barriers that offline models demand and increasing accessibility.

The findings found a correlation between first-time clients and both lower adherence and lost retention to PrEP KPLHS. As literature on enhanced counseling programs grows, targeting these interventions (such as LifeSteps) among new clients may best yield increased PrEP adherence. Facilitating these interventions with intensive follow up support (e.g. Information Communication and Technology (ICT) strategies and adherence feedback) may further enhance the effect of these programs [23].

Globally, TGW with HIV report suboptimal adherence and retention to HIV medication and have difficulty integrating it into their daily treatment regimen [24, 25]. The results of the multivariable analysis in this current study further supports the notion that TGW are a highly susceptible population to HIV treatment nonadherence and additional trans-focused efforts are needed. When approaching adherence support for TGW, the socio-cultural context around their lives must be considered. For example, a study reveals that potential PrEP and hormone interactions are barrier to PrEP adherence and retention for TGW, indicating the need for education addressing trans-specific concerns [26]. Furthermore, studies report that TGW do not feel comfortable accessing services designed for men and that disseminating PrEP information through MSM sources does not effectively reach TGW [26, 27]. Currently, the Princess PrEP program only has one CBO designated for TGW located in Pattaya City of Chonburi [28]. Thus, to provide adequate care for this population, the implementation of more TGW-specific programs is recommended. Taking limited resources into consideration, integrating additional adherence support marketed towards TGW into preexisting programs is an economical approach to potentially reducing barriers to care.

Among all the provinces, participants in Chonburi displayed the lowest rates of adherence. A possible explanation for poor adherence in this area is the culture of sex work and sex tourism in Pattaya’s prominent red-light district. The majority of participants from Chonburi were TGW who also engaged in sex work. Many of these participants were not Chonburi-native, and only traveled to the city during the months with large volume of tourists. Additionally, most of their clients were either from another city or country, and it is not uncommon that these individuals travel to another location for an extended period of time to engage in sex work. Being highly mobile could explain the low PrEP adherence among participants in Chonburi. Furthermore, the irregular time in which they meet up with the clients may also contribute to this phenomenon. Limited adherence research has been conducted on sex workers, a key population, which raises concerns on how to effectively provide PrEP outreach and support to this group [29]. In line with other effective sex worker HIV programs, an initial step towards improving the existing HIV prevention programs is applying methodical situation analyses to this area to determine the distribution and typologies of sex work (e.g. static versus mobile sex workers). Understanding the patterns of sex workers allows for targeted interventions based on context and location [30]. Some efforts utilizing community empowerment-based HIV programs, such as the notable one conducted in Sonagachi in India, have proven effective for improvements in prevention [29, 31, 32]. Therefore, PrEP services and support derived from these strategies serve as a potential step to providing proper care for sex workers. However, little research overall has been conducted on providing PrEP services for this population at higher risk of HIV acquisition. Additional research for this population is crucial, especially work that considers and differentiates the needs of male sex workers from TGW sex workers in HIV prevention care [29]. The use of the KPLHS which centers on the need of each specific key population could help address some of these serious concerns, including more research determining various modalities in delivering PrEP, including injectable and implant forms of PrEP.

This study does have several limitations. First and foremost, this research is done from the perspective of real world conditions from a resource limited, middle income country as a demonstration project; therefore, results were analyzed to maximize generalizability. The findings on PrEP adherence are not representative of populations outside of the CBOs in the study’s four provinces: Bangkok, Chiang Mai, Chonburi, and Songkhla. Given the feasibility of measuring PrEP adherence in a real-world setting, another major limitation is the reliance of self-reports for adherence. Studies have shown adherence self-reports are of low utility by themselves and recommend supplementing more reliable monitoring measures such as blood or hair assessments [33, 34]. As other data were also self-reported, various biases (social desirability, recall, etc.) may play a role, and the results may not accurately reflect participant behaviors and adherence to PrEP. Since this was a cross-sectional study taken at month 1 of the demonstration project, self-reported behaviors may not be reflective of long-term adherence. Additional assessments time points are necessary to make more casual inferences between the factors and dependent variables, as behaviors may change over time. Furthermore, the findings were estimated on an assumption that those who were considered as loss to follow-up had low adherence; therefore, the outcome from this study may not be applicable to settings in which high adherence was still observed despite care attrition.

## Conclusion

This current study is among the first to investigate adherence and retention patterns to PrEP among MSM and TGW accessing key population-led PrEP program in Thailand. The findings suggest that urgent, innovative strategies for PrEP adherence and retention support are needed, especially for vulnerable sub-populations such as younger users, transgender women, and new clients. Heading towards the next generation of preventative HIV care, increased efforts from civil society and government sectors will be crucial in providing effective prevention.

## Additional file

Additional file 1: Behavioral Risk Assessment Questionnaire. This questionnaire assessed the behavioral risk(s) that participants had. The answers were self-reported, and the participants completed the questionnaire by themselves with no guidance from the study staff. However, the participants were allowed to ask for clarification from the staff for the questions they did not understand or were uncertain. (PDF 111 kb)

## Abbreviations

CBO: Community-based organization
KPLHS: Key Population-Led Health Service
MSM: Men who have sex with men
PrEP: Pre-exposure prophylaxis
TGW: Transgender women
