# Supplementary material for: Correlates of nonadherence to key population-led HIV pre-exposure prophylaxis services among Thai men who have sex with men and transgender women
Source: BMC Public Health. 2019 Mar 21;19:328. doi: 10.1186/s12889-019-6645-0 (PMC6429797; doi:10.1186/s12889-019-6645-0)
Supplement: Supplementary file 1 — Behavioral Risk Assessment Questionnaire. This questionnaire assessed the behavioral risk(s) that participants had. The answers were self-reported, and the participants completed the questionnaire by themselves with no guidance from the study staff. However, the participants were allowed to ask for clarification from the staff for the questions they did not understand or were uncertain. (PDF 111 kb) [file 12889_2019_6645_MOESM1_ESM.pdf]

Date ☐☐-☐☐-☐☐☐☐ DD/MM/YYYY	PID ☐☐ - ☐☐☐☐
ACID: ☐☐☐☐☐☐☐☐☐	AC ID: ☐☐☐☐☐☐☐

Behavioral Risk Assessment Questionnaire (This questionnaire takes approximately 5 minutes to complete)

VISIT: ☐ screening ☐ month 3 ☐ month 6 ☐ month 9
☐ month 12 ☐ month 15 ☐ month 18 ☐ month 21
☐ month 24 ☐ month 27 ☐ month 30 ☐ month 33
☐ month 36

This is questionnaire, a recording data of clients services in Princess Somsawalee (Princess PrEP)

- You have the right not to answer any question in this questionnaire without any following adverse consequences. However, the answers you provide will help us understand more about your general characteristics.
- All information provided will be kept confidential and will be used for research purposes only. This information will not affect you personally or legally.
- Some questions may make you feel uncomfortable or is awkward to answer, which we would like to apologize in advance and thank you very much for sparing your time to complete this questionnaire.

Please mark an X in the box.

Questions 1-3 are for baseline visit only. Please skip to question #4.

1. How old were you when you first had sex? .... years old
2. In your LIFETIME, which gender(s) have you ever had sex with? (You may choose more than one answer)
   - ☐ Female
   - ☐ Male
   - ☐ Transgender woman
   - ☐ Prefer not to answer
3. Are you circumcised?
   - ☐ Yes, since .... years old
   - ☐ No
   - ☐ Prefer not to answer

   3.1 Have you ever taken PrEP in my life?
   - ☐ Yes
   - ☐ No

Date ☐☐-☐☐-☐☐☐☐ DD/MM/YYYY	PID ☐☐ - ☐☐☐☐
ACID: ☐☐☐☐☐☐☐☐☐	AC ID: ☐☐☐☐☐☐☐

If this is not a baseline visit, please start from question #4.

4. Within the past 3 months, which level of risk do you think you have for HIV acquisition?

☐ No risk

☐ Low risk

☐ Moderate risk

☐ High risk

5. Within the past 3 months, which gender(s) have you had sex with? (You may choose more than one answer)

☐ No sexual activity If your answer is "**no sexual activity**", please skip to question #13.

☐ Male, please specify number of male sex partners ....

☐ Female, please specify number of female sex partners ....

☐ Transgender woman, please specify number of transgender woman sex partners ....

6. Within the past 3 months, have you used condoms when having insertive or receptive anal sex?

☐ No

☐ Sometimes

☐ Always

☐ Prefer not to answer

6.1 Within the past 3 months, have you used condoms when you have receptive anal sex?

☐ No sexual activity with receptive anal sex within the past 3 months

☐ No

☐ Sometimes

☐ Always

☐ Prefer not to answer

6.2 Within the past 3 months, have you used condoms when you have insertive anal sex?

☐ No sexual activity with insertive anal sex within the past 3 months

☐ No

☐ Sometimes

Date ☐☐-☐☐-☐☐☐☐ DD/MM/YYYY	PID ☐☐ - ☐☐☐☐
ACID: ☐☐☐☐☐☐☐☐☐	AC ID: ☐☐☐☐☐☐☐

☐ Always

☐ Prefer not to answer

6.3 Within the past 3 months, have you used condoms when having sex with an HIV-positive partner?

☐Yes (next #6.4)

☐No (please skip to question #6.5)

☐Not sure of partner's HIV status (please skip to question #6.5)

6.4 If you have sex with an HIV-positive partner, do you know his/her viral load status?

☐Unknown

☐Partner has viral load detectable or partner does not receive ART

Please specify: how often do you use condoms with HIV-positive partner?

..... No

..... Sometimes

..... Always

..... Prefer not to answer

☐Partner has undetectable viral load

Please specify: How often do you use condoms with HIV-positive partner?

..... No

..... Sometimes

..... Always

..... Prefer not to answer

7. Within the past 3 months, have you used condoms when having insertive vaginal sex?

☐ No sexual activity with insertive vaginal sex within the past 3 months

☐No

☐ Sometimes

☐ Always

☐ Prefer not to answer

8. Within the past 3 months, have you used condoms when having receptive neovaginal sex? (If your answer is no, please skip to question #9)

Date ☐☐-☐☐-☐☐☐☐ DD/MM/YYYY	PID ☐☐ - ☐☐☐☐
ACID: ☐☐☐☐☐☐☐☐☐	AC ID: ☐☐☐☐☐☐☐

☐ No sexual activity with receptive vaginal sex within the past 3 months

☐ No

☐ Sometimes

☐ Always

☐ Prefer not to answer

9. Within the past 3 months, which addictive substances or stimulants have you used? (Please mark all that apply)

☐ Have not used any addictive substances/stimulants (Please skip to question #11)

☐ Alcohol

☐ Methamphetamine/Amphetamine (Yaba, Ice, Crystal Meth, Speed)

☐ Ecstasy (Ya E)

☐ Ketamine (Ya K)

☐ Poppers

☐ Heroin

☐ Cocaine

☐ Marijuana (Weed, Pot)

☐ LSD (Gra-dard Mao, Acid)

☐ Barbiturates and other sleeping pills/transquilizers

☐ Viagra or other drugs of the same type

☐ Not sure what substances I have used/ I have used other substances not included on the list

☐ Prefer not to answer

10. What is/are your purpose(s) for using these addictive substances/stimulants?

☐ To increase sexual arousal before and/or during sex

☐ To prolong an erection or prolong sexual activity

☐ To forget about one's sufferings or bad memories

☐ Wanted to try/Friends persuaded

☐ Addicted, needs to take it regularly

☐ Prefer not to answer

Date ☐☐-☐☐-☐☐☐☐ DD/MM/YYYY	PID ☐☐ - ☐☐☐☐
ACID: ☐☐☐☐☐☐☐☐☐	AC ID: ☐☐☐☐☐☐☐

11. Within the past 3 months, have you experienced any sign or symptom of sexually transmitted diseases or have you been diagnosed with sexually transmitted diseases such as gonorrhea, chalmydia, syphilis, and herpes (excluding warts)?

☐ No

☐ Yes

☐ Unsure

☐ Prefer not to answer

12. Within the past 3 months, where have you met your sex partners? (Please mark all that apply)

☐ Restaurant

☐ Spa

☐ Sauna

☐ Fitness Center

☐ Pub, bar, or disco tech

☐ Shopping Center

☐ Online website

☐ None of the above

13. Within the past 3 months or last visit, have you had group sex (party sex)?

☐ No

☐ Yes

- How often do you have group sex? ………… (times) within the past 3 months or last visit
- Which channel do you have group sex (party sex)? (You may choose more than one answer)

☐appoint by online ☐friends ☐Part of party

☐ Sauna ☐meet in pub, bar, or club

☐others ………

- During a group sex, how many partner(s) do you have sex with? ………… (person/people)
- Have you used condoms when having group sex?

☐No ☐ Sometimes ☐ Always

- Have you ever used substance(s) or stimulant(s) before or during sex?

☐Not yet ☐

- Within the past 3 months, which addictive substance(s) or stimulant(s) have you used? (Please mark all that apply)

Date ☐☐-☐☐-☐☐☐☐ DD/MM/YYYY	PID ☐☐ - ☐☐☐☐
ACID: ☐☐☐☐☐☐☐☐☐	AC ID: ☐☐☐☐☐☐☐

- ☐ Have not used any addictive substances/stimulants
- ☐ Methamphetamine/Amphetamine (Yaba, Ice, Crystal Meth, Speed)
- ☐ Ecstasy (Ya E)
- ☐ Ketamine (Ya K)
- ☐ Poppers
- ☐ Heroin
- ☐ Cocaine
- ☐ Marijuana (Weed, Pot)
- ☐ LSD (Gra-dard Mao, Acid)
- ☐ Barbiturates and other sleeping pills/transquilizers
- ☐ Viagra or other drugs of the same type
- ☐ Not sure what substances I have used/ I have used other substances not included on the list
- ☐ Prefer not to answer

14. Within the past 3 months, have you received or exchanged gift(s) or money for sex?

- ☐ No
- ☐ Yes
- ☐ Prefer not to answer

14.1 If yes, how often did you use condoms?

- ☐ Sometimes
- ☐ Always
- ☐ Prefer not to answer
